# Central (Aortic) Cannulation versus Peripheral (Axillary or Femoral) Cannulation in Acute Type A Aortic Dissections: A Meta-Analysis of Comparative Studies

**DOI:** 10.31083/j.rcm2505156

**Published:** 2024-05-06

**Authors:** Jiawang Ma, Hong Wang, Xiaomeng Wang, Xiaotong Hou

**Affiliations:** ^1^Center for Cardiac Intensive Care, Beijing Anzhen Hospital, Capital Medical University, 100054 Beijing, China

**Keywords:** type A aortic dissection, aortic cannulation, femoral cannulation, axillary cannulation, stroke, temporary neurological dysfunction

## Abstract

**Background:**

There has been an increased interest in using antegrade cannulation techniques during surgery for type A aortic dissection. While the utilization of central artery cannulation has been on the rise in recent times, its effectiveness and safety still require thorough examination. This study aimed to explore both the efficiency and safety of central arterial cannulation.

**Methods:**

A meta-analysis was conducted on studies that evaluated surgical outcomes when using central artery cannulation (CAC) in comparison to axillary artery cannulation (AXC) or femoral artery cannulation (FAC).

**Results:**

10 retrospective observational studies were included, enrolling 3022 patients (CAC = 1208 vs. FAC = 606; CAC = 1051 vs. AXC = 1119). Among these, 4 articles involved axillary artery cannulation, femoral artery cannulation, and central artery cannulation. Central cannulation was linked to decreased short-term mortality [odds ratio, 0.66, 95% confidence interval (CI) (0.48, 0.89), χ^2^ = 3.27, *p* = 0.007; $I^{2}$ = 0; *p* = 0.86] compared to femoral cannulation. Additionally, central cannulation was associated with a lower occurrence of temporary neurological dysfunction (TND) [odds ratio, 0.57, 95% CI (0.38, 0.85), χ^2^ = 0.88, *p* = 0.006; $I^{2}$ = 0%, *p* = 0.83] when compared with femoral cannulation. However, there was no statistical significance in mortality and TND between the central cannulation and axillary cannulation groups.

**Conclusions:**

This meta-analysis reveals that central cannulation surpasses femoral cannulation in lowering short-term mortality and the occurrence of TND among patients undergoing surgery for type A acute aortic dissection. However, central cannulation does not exhibit a higher mortality and TND compared to axillary cannulation.

## 1. Introduction

While advancements in surgical technology have led to a reduction in mortality from acute type A dissection in recent years, it still remains high. Cerebrovascular accidents and postoperative neurological complications are a significant concern in these patients. These complications are associated with increased perioperative mortality [1].

Currently, increasing attention has been placed on choice of cannulation to address perioperative neurologic complications during repair of type A aortic dissections. Determining the cannulation site is often based on the surgeon’s preference and expertise, the patient’s condition, along with vascular considerations. This complexity makes it challenging to establish a unanimous agreement regarding the optimal site for cannulation in type A aortic dissections.

Cannulation strategies can be broadly categorized into three groups: axillary artery cannulation (AXC), femoral artery cannulation (FAC), and central artery cannulation (CAC, involving direct cannulation of the ascending aorta or aortic arch). Among these, axillary artery and femoral artery cannulation, currently stand as the most frequently used approaches. However, there has recently been a growing interest in central artery cannulation [2].

Each of these three cannulation methods has its own set of advantages and disadvantages. The FAC method is associated with a high stroke rate and complications, including inadequate lower body perfusion and thromboembolism [3, 4]. AXC is not recommended for patients with unstable hemodynamics due to the increased time needed to establish cardiopulmonary bypass (CPB), low flow rates, and the potential for brachial plexus injury [5, 6]. Although CAC is rapid and efficient, there is concern regarding the potential for insertion into the false lumen [6]. Furthermore, because this cannulation method is not frequently employed, there is uncertainty regarding its overall safety.

The objective of this study was to analyze the short-term postoperative outcomes of central artery cannulation versus peripheral artery cannulation (FAC or AXC).

## 2. Methods

This study followed the guidelines outlined by the Prescribed Reporting Items in Systematic Reviews and Meta-analyses (PRISMA). This study has been registered at 88 PROSPERO and the registration number is CRD42023455546.

### 2.1 Literature Search Strategy

MEDLINE, EMBASE and Web of Science were searched up to April 6, 2023. Headings (MeSH) terms and EMTREE keywords: “Aortic Dissection”, “Aortic Dissecting Aneurysm”, “Catheterization”, “Cannulation”, etc. A total of 1445 references were retrieved, and 10 references were included in our study.

### 2.2 Selection of Articles

Two independent investigators reviewed all article titles and abstracts. The inclusion criteria included: (1) Studies involving both the axillary/subclavian artery and the central artery or both the femoral artery and the central artery (some articles may include the above three cannulation methods); (2) Include at least one primary endpoint; (3) Baseline characteristics of the population at the cannulation site should be available; (4) Each group should contain at least 10 patients. Non-English language, review articles, and comments were excluded. Studies of peripheral cannulation were also excluded when both axillary artery cannulation and femoral artery cannulation were consolidated into a single group.

### 2.3 Data Extraction and Literature Quality Assessment

Our meta-analysis focused on short-term mortality, cerebrovascular accidents (strokes), and neurological complications (TND, temporary neurological dysfunction) as primary endpoints. Data collection including the following items: study design, study date, study country, total number of patients, average age, cannulation site, surgical approach, duration of surgery, postoperative mortality, postoperative stroke, postoperative neurological complications, postoperative kidney issues, postoperative bleeding, and length of follow-up. In cases of disagreement, consensus was reached. The Newcastle-Ottawa scale was used to assess each of the included articles. This scale assigns a total score of 9 points, with scores exceeding 6 points indicating high-quality literature.

### 2.4 Statistical Analysis

Differences between the two groups was evaluated using odds ratios (ORs) and its 95% confidence interval (CI). Heterogeneity between studies was assessed using the χ^2^ test and the Cochrane Q score (reported as $I^{2}$, representing a percentage value of heterogeneity). If $I^{2}$ < 50%, the heterogeneity was not significant, and the fixed effect model was adopted. Otherwise, the random effects model was used. We first assessed possible sources of heterogeneity in selected studies. We used Review Manager (RevMan) [Computer program]. Version 5.4 (The Cochrane Collaboration, The Nordic Cochrane Centre, Copenhagen, Denmark) for data analysis. Data are expressed as mean ± standard deviation; For any test or model, a *p*-value of 0.05 was considered statistically significant.

### 2.5 Sensitivity and Publication Bias Analysis

**Fig. 1.**
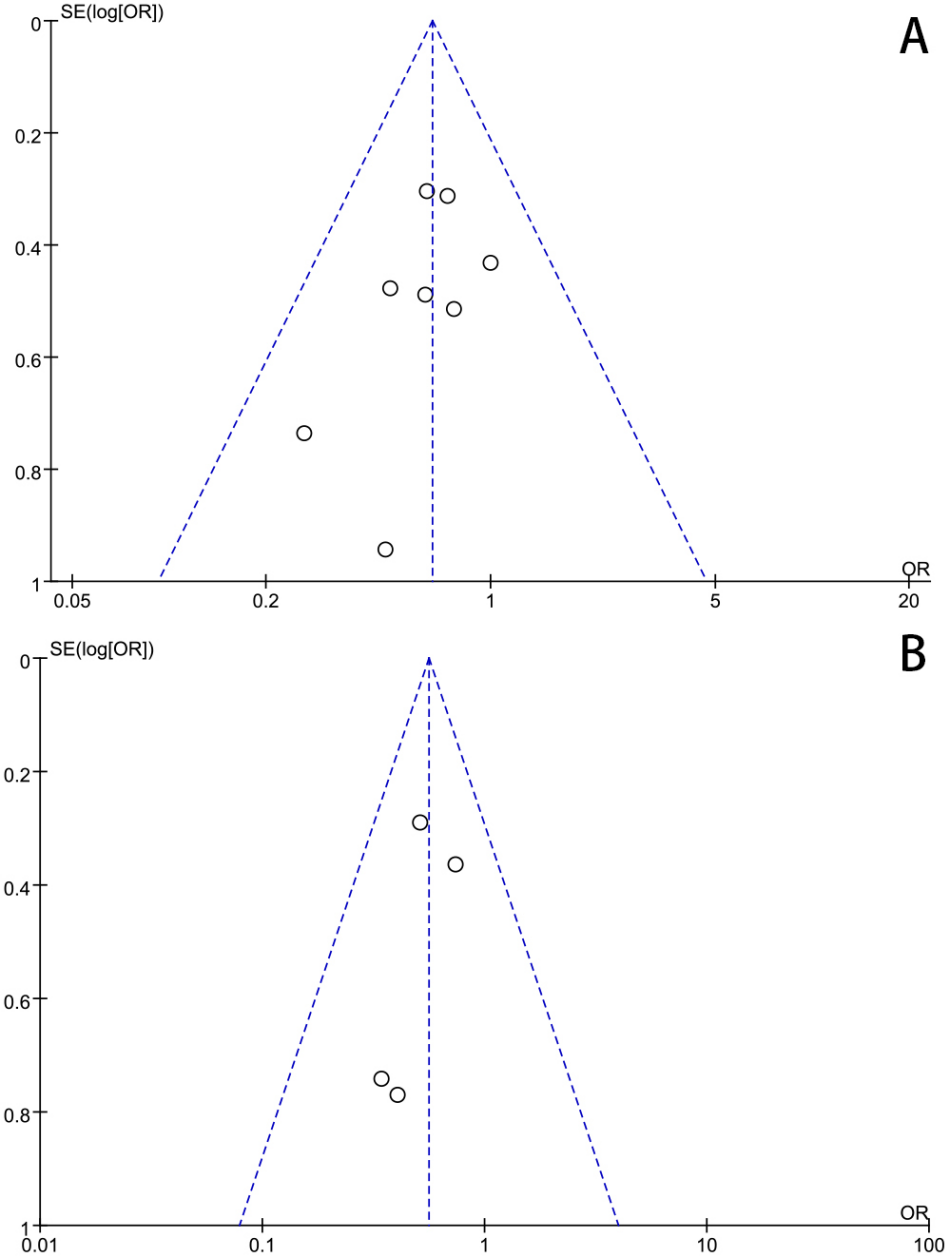
**Publication bias analysis by funnel plot graphic for the outcomes**. (A) Short-term mortality (CAC vs. FAC). (B) Neurological complications (CAC vs. FAC). ORs, odds ratios; FAC, femoral artery cannulation; CAC, central artery cannulation.

We excluded articles with the highest patient count or large odds ratios (ORs), and the outcomes remained largely consistent following data pooling, with no substantial changes. To assess publication bias, we utilized funnel plots, as depicted in Fig. 1, illustrating the mortality, stroke rates, and neurological complications within the FAC, AXC, and CAC groups. The funnel plot exhibited symmetry, implying the absence of publication bias in the study.

## 3. Results

**Fig. 2.**
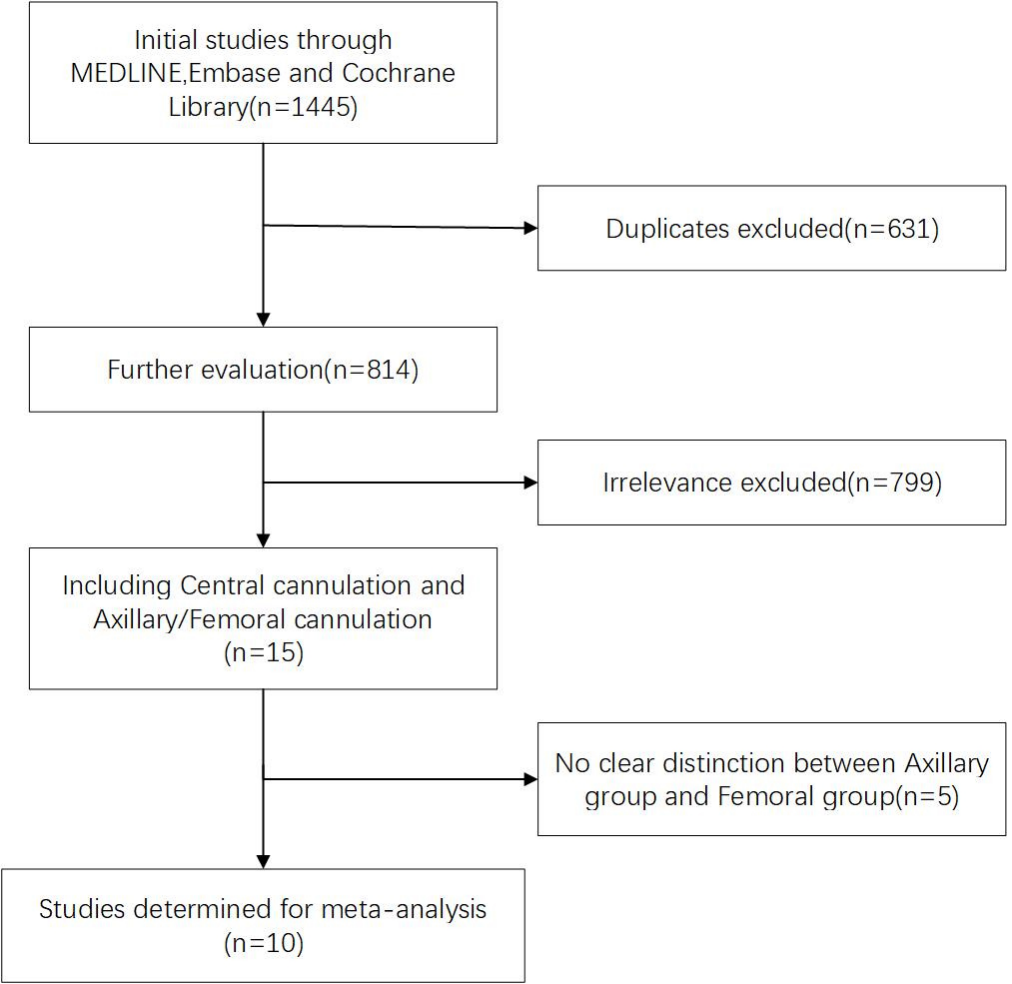
**Flowchart depicting study selection for meta-analysis**.

A total of 1445 articles were initially obtained. After assessing titles and abstracts in accordance with the inclusion criteria mentioned above, a final selection yielded 15 articles relevant to CAC. After in-depth reading and evaluation of the full texts, five articles that compared central cannulation with peripheral cannulation were excluded (as peripheral cannulation was not further categorized into axillary artery or femoral artery groups). Ultimately, a total of 10 articles meeting the criteria were included. Among these, eight were related to FAC, and six were related to AXC (4 of the 10 included both FAC and AXC). All 10 studies were retrospective in nature. In the meta-analysis, we separately analyzed CAC vs. FAC (1208 vs. 606) and CAC vs. AXC (1051 vs. 1119). Quality evaluation of the research, an overview of the research, and the process of selecting the final studies are presented in Tables 1,2 (Ref. [5, 6, 7, 8, 9, 10, 11, 12, 13, 14]) and Fig. 2, respectively. Article selection and quality assessment were independently conducted by two researchers, and each achieved high quality scores (>6). The preoperative, intraoperative and postoperative patient data are shown in Table 3A,3B,3C (Ref. [5, 6, 7, 8, 9, 10, 11, 12, 13, 14]) and Table 4 (Ref. [5, 6, 7, 8, 9, 10, 11, 12, 13, 14]).

**Table 1.** **Newcastle-Ottawa quality assessment scale**.

Author (year)	Selection				Comparability	Outcomes			Total
	1	2	3	4	5	6	7	8	
Sabashnikov *et al*. (2016) [7]	*	*	*		*	*	*	*	7
Rosinski *et al*. (2019) [14]	*	*	*		**	*			6
Norton *et al*. (2022) [6]	*	*	*		*	*	*	*	7
Suenaga *et al*. (2015) [12]	*	*	*		**	*	*	*	8
Ma *et al*. (2018) [8]	*	*	*		*	*	*	*	7
Kamiya *et al*. (2009) [9]	*	*	*		**	*	*	*	8
Kreibich *et al*. (2019) [5]	*	*	*		*	*	*	*	7
Yousef *et al*. (2022) [13]	*	*	*		*	*	*	*	7
Kusadokoro *et al*. (2020) [10]	*	*	*		*	*	*	*	7
Gegouskov *et al*. (2018) [11]	*	*	*		*	*	*	*	7

1, Clear criteria for grouping AXC/FAC/CAC; 2, Representative of AXC/FAC/CAC; 3, AXC/FAC/CAC patients were included from the same population; 4, No differences between three groups in terms of patient sex, age, type of surgery, or emergency surgery; 5, Indication for surgery; age and cerebral protection strategy; 6, Assessment of outcome; 7, Follow-up long enough; 8, Adequacy of follow-up of cohorts; * = grade.

**Table 2.** **Study overview**.

Author (year)	Design	Location	Opreative years	N			Age			Female (%)			U/E (%)	Comorbidities	FU
				AXC	FAC	CAC	AXC	FAC	CAC	AXC	FAC	CAC			
Sabashnikov *et al*. (2016) [7]	R	German	2006–2015	51	ND	17	69 (58, 74)	ND	70 (55, 77)	26 (51.0)	ND	9 (52.5)	ND	3, 4, 9	10 y
Rosinski *et al*. (2019) [14]	R	USA	2000–2017	617	93	65	61 ± 14	64 ± 13	61 ± 16	228 (37)	35 (38)	24 (37)	775 (100)	1–6, 9–11	ND
Norton *et al*. (2022) [6]	R	USA	2015–2020	192	ND	72	62 (53, 72)	ND	59 (49, 68)	76 (40)	ND	17 (24)	ND	1–3, 6, 9, 11	2.4 ± 1.6 y
Suenaga *et al*. (2015) [12]	R	Japan	2000–2013	ND	34	46	ND	74.5 ± 8.7	71.9 ± 11.7	ND	26 (76)	30 (65)	ND	1, 4, 5	6.8 y
Ma *et al*. (2018) [8]	R	China	2015–2017	ND	29	33	ND	47.90 ± 9.93	46.48 ± 10.32	ND	8 (27.59)	4 (12.12)	ND	1–3, 7	ND
Kamiya *et al*. (2009) [9]	R	German	1988–2007	ND	153	82	ND	57 ± 12	56 ± 14	ND	52 (34)	21 (26)	ND	ND	1, 5, 10, 15 y
Kreibich *et al*. (2019) [5]	R	USA	2006–2017	101	128	355	58 ± 14	60 ± 14	60 ± 14	31 (31)	45 (35)	133 (37)	ND	1–9	4.1 ± 3.1 y
Yousef *et al*. (2022) [13]	R	USA	2007–2021	54	33	490	60.4 ± 13.3	60.3 ± 12.3	61.5 ± 13.5	23 (42.6)	12 (36.4)	199 (40.6)	577 (100)	2, 3, 5, 9–11	4.76 y
Kusadokoro *et al*. (2020) [10]	R	Japan	1990–2018	104	104	52	62 (54–69)	64 (51–71)	63 (49–73)	38 (36)	44 (42)	21 (40)	364 (100)	1, 5, 6, 7, 9	5.8 ± 5.4 y
Gegouskov *et al. *(2018) [11]	R	Bulgaria	2008–2015	ND	32	85	ND	64.8 (46–79)	56.2 (22–81)	ND	8	26	117 (100)	1, 3, 5, 7–9	ND

y, year; R, retrospective; FU, follow-up; ND, not determined; N, number of patients; U/E, urgent/emergency surgery; AXC, axillary artery cannulation; FAC, femoral artery cannulation; CAC, central artery cannulation; Comorbidities: 1, hypertension; 2, COPD; 3, CAD; 4, neurological deficit; 5, malperfusion (cerebral, visceral and lower extremity); 6, renal failure; 7, Marfan syndrome; 8, Hyperlipidemia; 9, Diabetes mellitus; 10, Pericardial tamponade; 11, Peripheral vascular disease.

**Table 3A.** **Preoperative characteristics of eligible studies**.

Author(year)	Shock			Tamponade			Hemodynamic instability (Shock & Tamponade)		
	AXC	FAC	CAC	AXC	FAC	CAC	AXC	FAC	CAC
Sabashnikov *et al*. (2016) [7]	ND	ND	ND	ND	ND	ND	16	ND	5
Rosinski *et al*. (2019) [14]	15	11	6	49	28	11	89	34	21
Norton *et al*. (2022) [6]	19	ND	3	37	ND	4	56	ND	7
Suenaga *et al*. (2015) [12]	ND	8	18	ND	ND	ND	ND	8	18
Ma *et al*. (2018) [8]	ND	ND	ND	ND	ND	ND	ND	ND	ND
Kamiya *et al*. (2009) [9]	ND	37	9	ND	ND	ND	ND	37	9
Kreibich *et al*. (2019) [5]	17	39	107	18	33	79	35	72	186
Yousef *et al*. (2022)* [13]	17	9	156	Count with shock			17	9	156
Kusadokoro *et al*. (2020) [10]	26	31	14	ND	ND	ND	26	31	14
Gegouskov *et al*. (2018) [11]	ND	2	11	ND	ND	ND	ND	2	11

*Yousef’s study put shock, tamponade, and rupture together as one variable.

**Table 3B.** **Intraoperative characteristics of eligible studies**.

Author(year)	Surgical procedure	CPB time (min)			ACC time (min)			HCA time (min)			Operation time (min)		
		AXC	FAC	CAC	AXC	FAC	CAC	AXC	FAC	CAC	AXC	FAC	CAC
Sabashnikov *et al*. (2016) [7]	1–6	174 (130; 234)	ND	194 (118; 298)	85 (65; 130)	ND	111 (67; 164)	30 (18; 47)	ND	56 (16; 78)	322 (247; 420)	ND	311 (244; 426)
Rosinski *et al*. (2019) [14]	1–6	159 ± 58	148 ± 57	157 ± 64	94 ± 46	85 ± 44	99 ± 50	0/20/36	9/23/36	0/13/29	ND	ND	ND
Norton *et al*. (2022) [6]	1–6	222 (184, 279)	ND	200 (163, 251)	150 (113, 204)	ND	144 (109, 181)	28 (22, 40)	ND	28 (18, 43)	ND	ND	ND
Suenaga *et al*. (2015) [12]	ND	ND	148 ± 20	141 ±17	ND	75 ± 17	66 ± 15	ND	32 ± 5.5	32 ± 7.8	ND	249 ± 47	221 ± 29
Ma *et al*. (2018) [8]	1–6	ND	298.28 ± 95.89	260.97 ± 45.14	ND	193.55 ± 57.97	170.67 ± 41.72	ND	37.00 ± 9.39	40.97 ± 7.98	ND	536 ± 155	440 ± 68
Kamiya *et al*. (2009) [9]	1–5	ND	206 ± 95	218 ± 105	ND	105 ± 55	105 ± 45	ND	17 ± 24	20 ± 20	ND	332 ± 138	357 ± 139
Kreibich *et al*. (2019) [5]	1–5	212 (176–252)	212 (181–254)	198 (167–238)	131 (105–173)	148 (112–179)	125 (103–160)	36 (27–49)	35 (28–55)	32 (25–42)	379 (310–460)	323 (283–403)	316 (264–378)
Yousef *et al*. (2022) [13]	1–6	239 ± 86.8	217 ± 68.0	200 ± 71.4	166 ± 65.1	149 ± 67.1	136 ± 59.8	24.1 ± 24.9	14.6 ± 17.5	12.5 ± 20.9	ND	ND	ND
Kusadokoro *et al*. (2020) [10]	1–6	133 (113–169)	138 (115–187)	155 (127–212)	94 (81–120)	90 (70–118)	102 (82–133)	ND	ND	ND	360 (320–469)	340 (270–435)	323 (254–425)
Gegouskov *et al*. (2018) [11]	1–6	ND	176 (87–323)	155 (78–288)	ND	143 (57–225)	123 (44–207)	ND	31 (22–47)	27 (9–73)	ND	324 (181–808)	297 (164–733)

**Table 3C.** **Intraoperative brain protection of eligible studies**.

Author(year)	ACP			RCP			ACP & RCP			Lowest temperature		
	AXC	FAC	CAC	AXC	FAC	CAC	AXC	FAC	CAC	AXC	FAC	CAC
Sabashnikov *et al*. (2016) [7]	51	ND	17	ND	ND	ND	ND	ND	ND	all MHCA		
Rosinski *et al*. (2019) [14]	51	1	6	409	66	30	12	1	2	ND	ND	ND
Norton *et al*. (2022) [6]	183	ND	33	0	ND	21	6	ND	13	22 (18, 25)	ND	23 (19, 25)
Suenaga *et al*. (2015) [12]	ND	ND	ND	ND	ND	ND	ND	ND	ND	all 25 °C		
Ma *et al*. (2018) [8]	ND	29	33	ND	ND	ND	ND	ND	ND	ND	26.05 ± 2.78	25.49 ± 2.07
Kamiya *et al*. (2009) [9]	ND	14	11	ND	0	0	ND	0	0	ND	24.6 ± 4.9	24.4 ± 5.9
Kreibich *et al*. (2019) [5]	63	4	65	29	116	264	9	8	26	ND	ND	ND
Yousef *et al*. (2022) [13]	ND	ND	ND	ND	ND	ND	ND	ND	ND	ND	ND	ND
Kusadokoro *et al*. (2020) [10]	all ACP			ND	ND	ND	ND	ND	ND	all 20~25 °C		
Gegouskov *et al*. (2018) [11]	ND	27	81	ND	ND	ND	ND	ND	ND	all 26~30 °C		

CPB, cardiopulmonary bypass; ACC, aortic cross-clamp; ACP, antegrade cerebral perfusion; RCP, retrograde cerebral perfusion; AXC, axillary artery cannulation; FAC, femoral artery cannulation; CAC, central artery cannulation; ND, not determined; MHCA, moderate hypothermic circulatory arrest; HCA, hypothermic circulatory arrest. Surgical procedure: 1, ascending aortic replacement; 2, hemi-arch replacement; 3, total arch replacement; 4, root replacement; 5, aortic valve replacement; 6, other procedures (CABG, descending aortic replacement, elephant trunk procedures and mitral valve replacement).

**Table 4.** **Postoperative outcomes and complications of eligible studies**.

Author (year)	N			Short-term mortality			Cerebrovascular accident (Stroke)			Neurological complications (TND)			Renal failure or CRRT			Bleeding		
	AXC	FAC	CAC	AXC	FAC	CAC	AXC	FAC	CAC	AXC	FAC	CAC	AXC	FAC	CAC	AXC	FAC	CAC
Sabashnikov *et al*. (2016) [7]	51	ND	17	16	ND	13	7	ND	3	17	ND	4	9	ND	2	13	ND	1
Rosinski *et al*. (2019) [14]	617	93	65	45	15	7	47	8	9	ND	ND	ND	53	12	8	52	11	6
Norton *et al*. (2022) [6]	192	ND	72	21	ND	5	10	ND	4	1	ND	0	14	ND	8	6	ND	2
Suenaga *et al*. (2015) [12]	ND	34	46	ND	3	2	ND	6	5	ND	5	3	ND	3	1	ND	0	7
Ma *et al*. (2018) [8]	ND	29	33	ND	8	3	ND	1	3	ND	ND	ND	ND	3	2	ND	0	1
Kamiya *et al*. (2009) [9]	ND	153	82	ND	54	21	ND	7	4	ND	31	13	ND	18	10	ND	ND	ND
Kreibich *et al*. (2019) [5]	101	128	355	11	17	36	10	15	46	8	23	36	13	12	50	10	19	36
Yousef *et al*. (2022) [13]	54	33	490	8	6	48	3	1	18	ND	ND	ND	6	5	55	5	4	40
Kusadokoro *et al*. (2020) [10]	104	104	52	22	20	10	5	6	6	ND	ND	ND	2	1	3	8	9	2
Gegouskov *et al*. (2018) [11]	ND	32	85	ND	7	15	ND	3	3	ND	6	9	ND	3	7	ND	2	4

TND, temporary neurological dysfunction; ND, not determined; CRRT, continuous renal replacement therapy; AXC, axillary artery cannulation; FAC, femoral artery cannulation; CAC, central artery cannulation; N, number of patients.

### 3.1 Short-Term Mortality

**Fig. 3.**
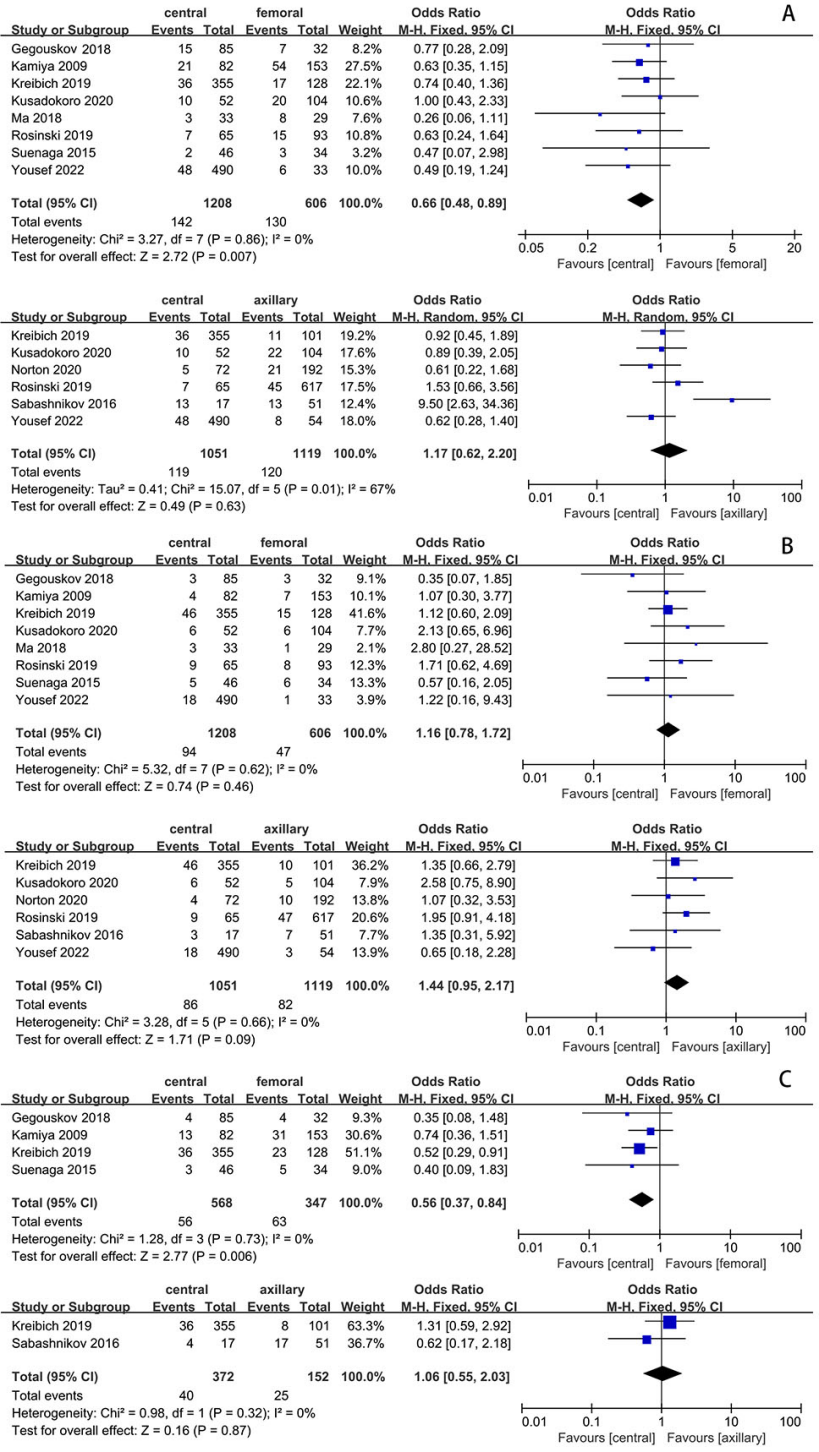
**Comparison of outcomes of interest between CAC vs. AXC & CAC vs. FAC**. (A) Short-term mortality. (B) Cerebrovascular accident. (C) Neurological complications (TND). CAC, central artery cannulation; AXC, axillary artery cannulation; FAC, femoral artery cannulation; TND, temporary neurological dysfunction.

A comparison of mortality between the FAC group and the CAC group was performed in eight studies. The results of the meta-analysis indicated that the combined mortality rate was 21.5% (130/606) in the FAC group and 11.8% (142/1208) in the CAC group. This is shown in Fig. 3A, where the differences in mortality achieved statistical significance [odds ratio, 0.66, 95% CI (0.48, 0.89), χ^2^ = 3.27, *p* = 0.007]. The test for heterogeneity showed no significant heterogeneity ($I^{2}$ = 0; *p* = 0.86), suggesting the validity of data pooling.

Mortality between the AXC group and the CAC group were investigated in six studies. The combined mortality rate was 10.7% (120/1119) in the AXC group and 11.3% (119/1051) in the CAC group. However, the meta-analysis results revealed no statistical significance between the two groups, as indicated in Fig. 3A [odds ratio, 1.17, 95% CI (0.62, 2.20), χ^2^ = 15.07, *p* = 0.63; $I^{2}$ = 67%, *p* = 0.01].

### 3.2 Cerebrovascular Accident (Stroke)

There were a total of eight studies that provided a comparison of stroke rates between the FAC group (7.8%, 47/606) and the CAC group (7.8%, 94/1208). Additionally, six studies reported the comparison of stroke rates between the AXC group (7.3%, 82/1119) and the CAC group (8.2%, 86/1051), as shown in Fig. 3B. Notably, none of the results demonstrated statistical significance. The meta-analysis findings indicate that the incidence of postoperative stroke in the CAC group exhibited no discernible difference compared to the AXC group and the FAC group.

### 3.3 Neurological Complications (TND)

Postoperative TND were reported in 4 articles for both the FAC group and the CAC group. The combined rates of neurological complications were 18.7% (65/347) in the FAC group and 10.7% (61/568) in the CAC group. The results of the meta-analysis showed statistically significant differences in the incidence of neurological complications between the two groups Fig. 3C [odds ratio, 0.56, 95% CI (0.37, 0.84), χ^2^ = 1.28, *p* = 0.006; $I^{2}$ = 0%, *p* = 0.73], which indicates that the pooling of the data was valid.

In contrast, two studies [5, 7] presented a comparison of postoperative neurological complication rates between the AXC group (16.4%, 25/152) and the CAC group (10.8%, 40/372). However, the pooled data analysis, as depicted in Fig. 3C, revealed no statistical significance [odds ratio, 1.06, 95% CI (0.55, 2.03), χ^2^ = 0.98, *p* = 0.87; $I^{2}$ = 0%, *p* = 0.32].

### 3.4 Secondary Endpoints

**Fig. 4.**
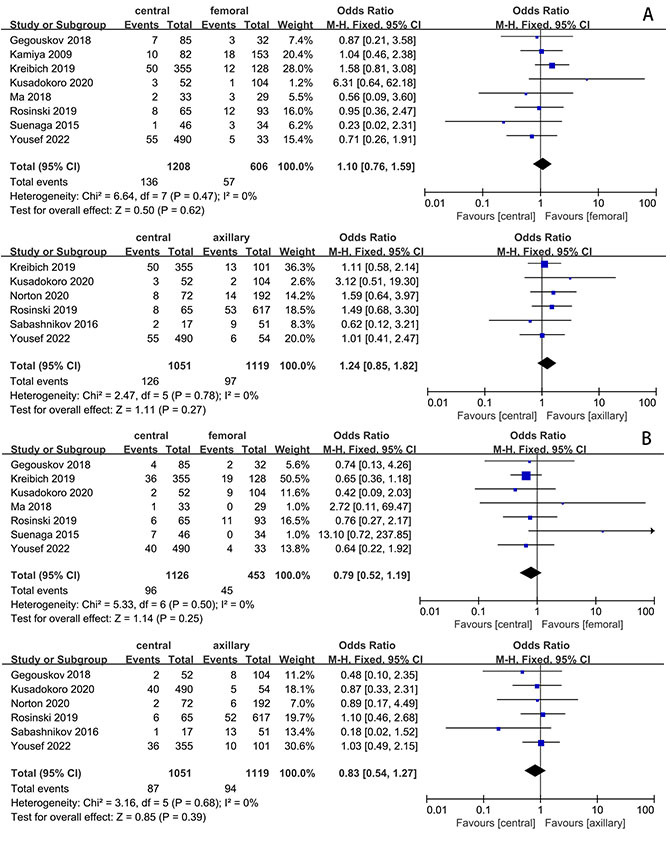
**Comparison of outcomes of interest between CAC vs. AXC & CAC vs. FAC**. (A) Renal failure or continuous renal replacement therapy (CRRT). (B) Bleeding. CAC, central artery cannulation; AXC, axillary artery cannulation; FAC, femoral artery cannulation.

We also conducted an analysis of the incidence of postoperative renal failure or continuous renal replacement therapy (CRRT), as well as reoperations for bleeding. However, no significant differences were observed in these endpoints, as depicted in Fig. 4A,B.

## 4. Discussion

This meta-analysis assessed the short-term outcomes of central artery cannulation in comparison to two conventional peripheral cannulation methods. Given that central artery cannulation is not commonly employed in most centers, this study undertook a comparison between the CAC group and the FAC group, as well as between the CAC group and the AXC group. The objective was to determine the surgical outcomes of the CAC group when using these two traditional cannulation techniques at individual centers. The findings demonstrated that, in terms of short-term mortality, CAC outperformed FAC. Moreover, there existed no significant distinction in outcomes between CAC and AXC. Additionally, the incidence of postoperative TND was lower with CAC compared to FAC.

Following rigorous screening by two independent researchers, a total of 10 studies were incorporated into this meta-analysis. The collective dataset encompassed 3022 patients drawn from these 10 studies. We found that patients with a type A aortic dissection may significantly benefit from CAC when compared to FAC, by achieving a marked reduction in mortality as well as the incidence of TND. Retrograde flow from femoral artery cannulation could potentially increase perfusion of the false lumen and contribute to multi-organ failure and thromboembolism [15, 16]. In contrast to the FAC approach, CAC offers distinct benefits. By circumventing retrograde blood flow, it mitigates the risk of embolization triggered by plaque detachment, and limits false lumen perfusion.

In a comparison of CAC and AXC, patients in both groups showed similar rates of mortality, cerebrovascular accident, and TND. However, the biggest drawback of AXC is time, the time-intensive process of isolating the axillary artery. This has increased interest for a more direct cannulation approach, particularly in cases involving patients with hemodynamic instability. CAC is increasingly gaining traction across various medical centers to address such scenarios [17]. Notably, this approach obviates the necessity for supplementary skin incisions and extensive axillary artery dissection, expedites the establishment of CPB and decreases operative times. When dealing with patients with cardiac tamponade or hemodynamic instability, the initiation of CPB through this strategy is more expeditious to avoid perioperative complications. This technique rapidly achieves perfusion of the true lumen that can prove particularly advantageous for patients with the malperfusion syndrome [18].

In our review of 10 studies, the use of CAC varied among centers. Dr. Etsuro Suenaga and Dr. Sarah Yousef’s facilities preferred CAC [12, 13], while other centers considered factors like patient hemodynamic stability and the feasibility of axillary artery cannulation. However, surgeon preference emerged as the primary determining factor. In Brad F. Rosinski’s study [14], focusing on hemodynamic instability scenarios such as shock, new heart failure, cardiac arrest, rupture, or tamponade, the use of femoral or central artery cannulation prevailed over axillary artery cannulation. The rationale was to rapidly alleviate pressure on the patient’s aorta by promptly opening the pericardium [14]. Yet, our comprehensive analysis of data related to shock, tamponade, and other conditions across the 10 studies did not yield a consistent conclusion (**Supplementary Fig. 1**). This inconsistency may be attributed to surgeon preferences—despite preferences for CAC in specific centers, it’s noteworthy that the majority of surgeons still lean towards traditional peripheral arterial cannulation over CAC. In all 10 studies, without exception, preoperative aortic computed tomography angiography (CTA), trans esophageal echocardiography (TEE), and Seldinger cannulation were consistently employed, owing to advancements in imaging and cannulation techniques. In short, post-median sternotomy, a meticulous comparison between TTE and preoperative aortic CTA was conducted to ascertain the position of the lumen and the relationship between the true lumen and the false lumen. For patients undergoing a mediansternotomy for the first time, locating the true lumen at the distal end of the ascending aorta and the aortic arch is typically straightforward. Guided by intraoperative TEE, the distal true lumen from the puncture site can be accurately identified, ensuring consistent placement of the cannula in the true lumen [14]. The development of the Seldinger cannulation technique effectively reduced the risk of CAC potentially leading to aortic rupture or insertion into the false lumen [5, 6, 19].

The position of the cannula is not always placed in one site. One study looked into the repositioning of cannulation sites, revealing that approximately 11% of patients underwent a switch in cannulation position [14]. The most common transition occurred from FAC to CAC during the cooling phase, driven by surgeon preference or the identification of elevated blood flow resistance during cardiopulmonary bypass. Of the patients initially treated with CAC, 6 patients (9%) switched the cannulation position, 1 was switched to AXC due to aortic rupture, and 1 was switched to AXC due to high resistance on CPB and 1 was switched to AXC+FAC due to increased flow in false lumen, and the other 3 cases were Surgeon preference. It is crucial to underscore that the decision to change the cannulation site relies on the patient’s condition at the time and the surgeon’s thorough evaluation of perfusion blood flow. Given the study’s limited sample size, a cautious approach is recommended when considering a switch in cannulation site [14].

The choice of the arterial cannulation site can be influenced by strategies for cerebral protection. Surgeons opting for AXC often employ unilateral or bilateral anterograde cerebral perfusion. In cases involving aortic cannulation, attaining unilateral anterograde cerebral perfusion is typically achieved through innominate artery or common carotid artery cannulation. This often necessitates an additional arterial cannulation to ensure complete cerebral perfusion, with the direction of blood flow mirroring that of axillary artery perfusion [6]. Furthermore, the limitation imposed by the diameter of the femoral artery can impede adequate blood supply to the upper limbs and brain. This limitation may arise from the small diameter of FAC or the diversion of blood into the false lumen. A notable outcome of this study was the observation of a heightened incidence of TND following FAC (*p* = 0.006). This may be linked to instances of intraoperative cerebral hypoperfusion due to the aforementioned factors. However, no significant disparity in the postoperative stroke rate emerged between the two groups.

However, it is important to emphasize that this study does not advocate for central artery cannulation as the primary or preferred choice in all scenarios. Quite the opposite, our intention is to convey that central artery cannulation serves as a valuable alternative when circumstances make axillary artery cannulation challenging. Instances where axillary artery cannulation may prove difficult include cases of dissection extending to the axillary artery, situations where the axillary artery’s dimensions are inadequate to support optimal blood perfusion, and when axillary artery atherosclerosis poses a heightened risk of iatrogenic injury and hemodynamic instability—particularly in the context of proximal aortic surgery. For patients facing more intricate aortic dissection scenarios, such as total arch replacement, axillary artery cannulation remains the preferred approach, particularly among patients exhibiting relative stability. It is our belief that there exists no universally ideal cannulation method. Instead, it frequently necessitates consideration of several factors including the vascular, the location and extent of the dissection flap, the patient’s hemodynamic condition, and the presence of multi-system dysfunction.

A notable limitation of this study arises from its reliance on retrospective analyses, which often translates to arterial cannulation site choices being influenced by surgeons’ preferences, the severity of acute type A aortic dissection patients, the location of tears, and various other factors that collectively shape distinct surgical strategies. This inherent variability in surgical planning stemming from cannulation location may introduce a degree of bias into the postoperative outcomes. Furthermore, it is crucial to underscore that the data included in this study were amassed from specialized centers with extensive aortic surgical expertise. As such, caution should be exercised when extrapolating the conclusions to smaller or less experienced centers, which could potentially yield different outcomes.

## 5. Conclusions

Our study underscores the safety of central artery cannulation in patients with acute type A aortic dissection. When juxtaposed with the traditional femoral artery cannulation, it emerges as a significantly superior approach, effectively reducing short-term mortality rates and mitigating the incidence of neurological complications. Equally noteworthy, when compared to axillary artery cannulation, central artery cannulation exhibited comparable rates of mortality and postoperative complications. In summary, central artery cannulation is as a viable and beneficial alternative for patients with a type A aortic dissection when axillary artery cannulation is not feasible.

## Data Availability

The data supporting the findings of this study can be found within the article. Raw data can be obtained from the corresponding author upon a reasonable request.

## Abbreviations

CAC, central artery cannulation; AXC, axillary artery cannulation; FAC, femoral artery cannulation; TND, temporary neurological dysfunction; CPB, cardiopulmonary bypass; CI, confidence interval; ORs, odds ratios; CRRT, continuous renal replacement therapy.

## Supplementary Material

Supplementary material associated with this article can be found, in the online version, at https://doi.org/10.31083/j.rcm2505156.
