# Supplementary figures and images for: Central (Aortic) Cannulation versus Peripheral (Axillary or Femoral) Cannulation in Acute Type A Aortic Dissections: A Meta-Analysis of Comparative Studies
Source: Rev Cardiovasc Med. 2024 May 6;25(5):156. doi: 10.31083/j.rcm2505156 (PMC11267197; doi:10.31083/j.rcm2505156)

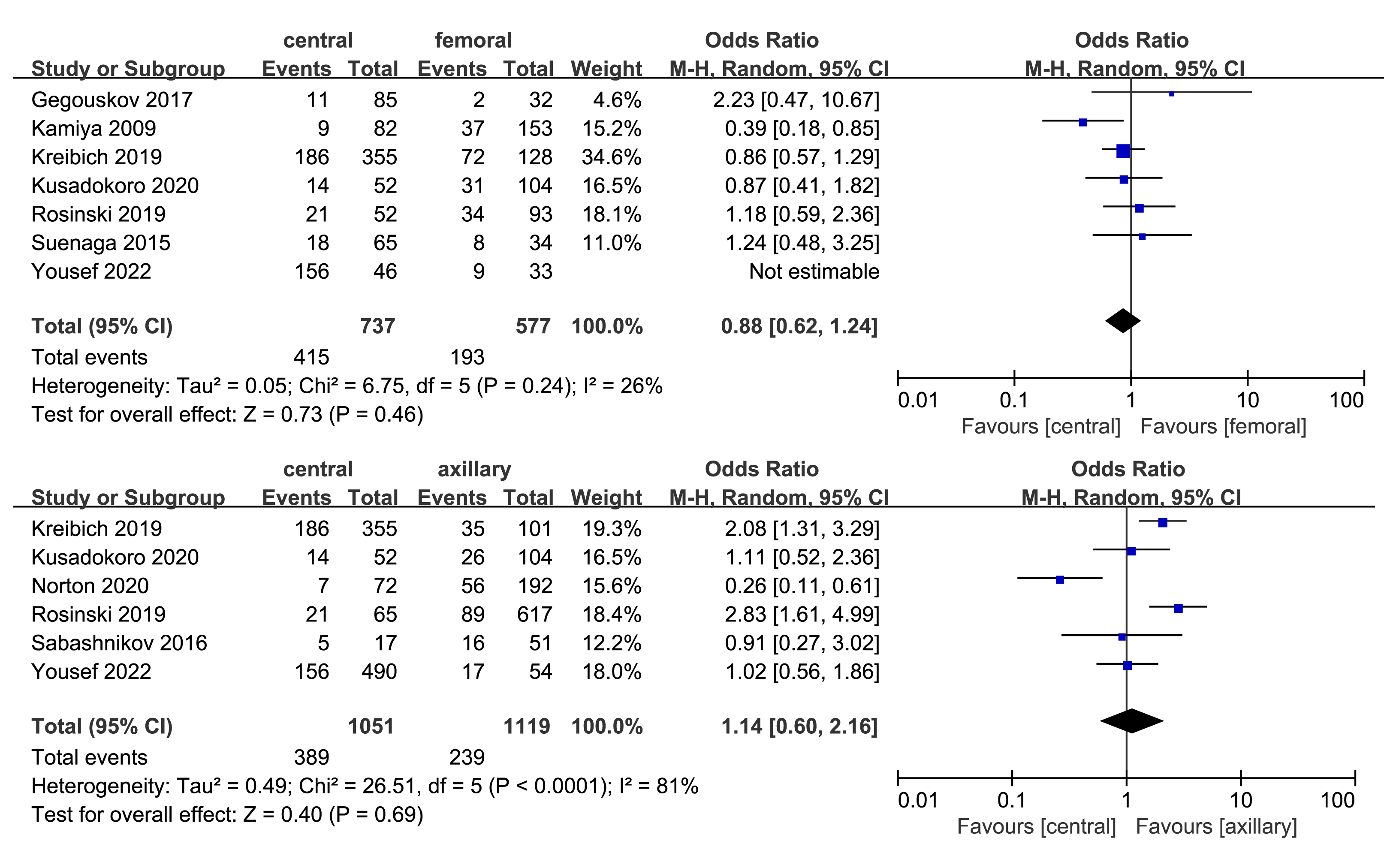

Supplement: Supplementary file 1 [file 2153-8174-25-5-156-s1.zip › 2153-8174-25-5-156-s1/Supplementary Fig. 1.tif]
